# Influence of Abiotic Stress Factors on the Antioxidant Properties and Polyphenols Profile Composition of Green Barley (*Hordeum vulgare* L.)

**DOI:** 10.3390/ijms21020397

**Published:** 2020-01-08

**Authors:** Przemysław Łukasz Kowalczewski, Dominika Radzikowska, Eva Ivanišová, Artur Szwengiel, Miroslava Kačániová, Zuzanna Sawinska

**Affiliations:** 1 Institute of Food Technology of Plant Origin, Poznań University of Life Sciences, 31 Wojska Polskiego St., 60-624 Poznań, Poland; artur.szwengiel@up.poznan.pl; 2 Department of Agronomy, Poznań University of Life Sciences, 11 Dojazd St., 60-632 Poznań, Poland; dominika.radzikowska@up.poznan.pl (D.R.); zuzanna.sawinska@up.poznan.pl (Z.S.); 3 Department of Technology and Quality of Plant Products, Slovak University of Agriculture in Nitra, 2 Tr. A. Hlinku St., 949 76 Nitra, Slovakia; eva.ivanisova@uniag.sk; 4 Department of Fruit Sciences, Viticulture and Enology, Slovak University of Agriculture in Nitra, 2 Tr. A. Hlinku St., 949 76 Nitra, Slovakia; miroslava.kacaniova@gmail.com; 5 Department of Bioenergy and Food Technology, Faculty of Biology and Agriculture, University of Rzeszów, 4 Zelwerowicza St., 35-601 Rzeszow, Poland

**Keywords:** barley grass, drought, solar radiation, flavonoids, phenolic acids, antioxidant activity, antimicrobial activity

## Abstract

The influence of stress factors on the plant can, on the one hand, lead to worse functioning of the plant and loss of its crop, but on the other, it can have a positive effect on the metabolism of compounds with documented biological activity. In this study, the effect of light and drought intensity on photosynthetic activity and physiological status of two barley varieties, as well as the antimicrobial, antioxidant properties and profile of polyphenolic compounds of green barley were analysed. It was shown that under the conditions of water shortage, the *KWS Olof* variety showed a smaller decrease in CO_2_ assimilation and transpiration and higher values of these parameters at both light intensities. Only in the *KWS Olof* variety increased stress as a result of increased light intensity. It has also been shown that both the intensity of radiation and drought-related stress have a significant impact on the profile of polyphenolic compounds from green barley, without a simple relationship between the impact of stress factors on the content of polyphenols. Changes in the profile of polyphenolic compounds augmented the antioxidant and antimicrobial activity of the material. This, in turn, proposes the possibility of reducing the applied doses of herbal material thanks to a greater content of active substances in extracts obtained from the plants used to produce medicinal preparations.

## 1. Introduction

Proper functioning of the human body depends on many factors, including the maintenance of the redox balance, which means that the reactive oxygen species (ROS), generated in metabolic processes, have to be eliminated [1]. ROS take part in the oxidation of lipids, proteins and even nucleic acids that lead to changes in cells or their death. Accumulation of a large quantity of ROS results in oxidative stress and this may eventually lead to diseases [2]. Nowadays, food is intended not only for nutrition, but also for the prevention of chronic and nutrition-related diseases, as well as for improving people’s general well-being. In connection with the growing knowledge of consumers about functional food products, new, functional raw materials are being required that contain appropriate phytochemical compounds that help fight diseases or oxidative stress [3,4,5]. One of these raw materials is green barley.

The term ‘green barley’ (GB) in the literature is used to describe barley seedlings (*Hordeum vulgare* L.), growing up to 200 h from germination, that reach about 20–30 cm in height [6]. Other terms used are young barley, barley shoots or barley grass. Young green barley leaves are a good natural source of vitamins and minerals. Research has shown that its composition includes 2-O-glycosyl isovitexin (2-O-GIV), flavone C-glycosides, saponarin, lutonarin, superoxide dismutase (SOD), catalase (CAT), peroxidase (POX), carotenoids, flavonoids, chlorophyll, as well as vitamins C and E [7,8]. GB also exhibits physiological activities, including hypolipidemic, antidiabetic, anti-ulcer effects and even anti-stress properties [9].

Plants are well-known sources of antioxidant compounds [10,11,12,13]. The amount of individual biologically active compounds in plants depends, however, on climate conditions, soil conditions and stress factors that may increase their content [14,15]. Stress factors can be divided into biotic and abiotic. Biotic (biological) stressors include harmful effects of other plants, viruses, bacteria, fungi and the occurrence of pests [16], while abiotic (physical) stressors include a deficiency, excess or incorrect intensity of UV radiation, extreme positive or negative temperatures, salinity, frosts, droughts, excess water, damage caused by snow, wind or lightning [17,18,19]. Through different mechanisms induced by abiotic stresses, plants produce different antioxidants, either non-enzymatic or enzymatic. Enhancement of health-promoting properties of plant origin foods will add value and create new opportunities for food producers.

Therefore, the aim of this study was to determine the effect of exposure to two abiotic stress factors, seven-day drought and intense solar radiation, on the content and composition of polyphenols in the green barley. The relationship between the stress factors and antioxidant activity of barley grass was analysed as well.

## 2. Results and Discussion

### 2.1. Effects of Abiotic Atresses on the Physiological Properties of GB

The first and basic physiological response of plants to drought stress is decreased in photosynthetic efficiency as a result of the stomata closing. This process is initiated within 1–2 min after the occurrence of the causative agent, and completed within 5 min. Maintaining the balance between CO_2_ exchange and transpiration is necessary to maximize CO_2_ assimilation in photosynthesis and, at the same time, reduce water loss [20].

There were significant differences in the photosynthesis rate (parameter A) between the varieties, within the light intensity and under the influence of drought (Table 1). The highest photosynthesis intensity expressed by CO_2_ assimilation was found in control plants at a light intensity of PAR = 800 µmol m^−2^ s^−1^, with the *Nokia* variety having higher photosynthetic activity than *KWS Olof*. Control plants growing at PAR = 400 µmol m^−2^ s^−1^ light intensity showed significantly lower photosynthesis activity for the *Nokia* variety by 24.1%, while for the *KWS Olof* variety by 20.3%. Similarly, at PAR = 800 µmol m^−2^ s^−1^, plants of the *Nokia* variety were characterized by higher intensity of CO_2_ assimilation compared to the *KWS Olof* variety.

Abiotic stress in the form of water deficiency in both varieties caused a significant decrease in CO_2_ assimilation, however at a different level. In the *Nokia* variety, the decrease in CO_2_ assimilation due to drought was 91.6% at PAR = 800 µmol m^−2^ s^−1^ and 87.8% at PAR = 400 µmol m^−2^ s^−1^, while in *KWS Olof* by 67.6% and 18.4%, respectively. In the *KWS Olof* variety, a much smaller decrease in CO_2_ assimilation as a result of drought was observed, while stress was intensified by increasing the intensity of PAR. The *KWS Olof* variety was characterized by lower CO_2_ assimilation under control conditions, but retained higher in drought stress. Smaller, but significant differences between varieties were noted in the transpiration level (parameter E). The highest water evaporation was observed in control plants at PAR = 800 µmol m^−2^ s^−1^, while the reduction of transpiration as a result of lowering PAR was 18.3% for the *KWS Olof* variety, and 26.1% for *Nokia*. Similarly to the intensity of CO_2_ assimilation, in the *KWS Olof* variety the decrease in transpiration as a result of drought was smaller (at PAR = 800 µmol m^−2^ s^−1^ by 78.3%, at PAR = 400 µmol m^−2^ s^−1^ by 35.3%) than in *Nokia* (at PAR = 800 µmol m^−2^ s^−1^ by 90, 6%, at PAR = 400 µmol m^−2^ s^−1^ by 84.2%). 

Stress-reduced CO_2_ assimilation can lead to an imbalance between the supply and demand of ATP and NADPH assimilation strength in photosynthesis. In such a situation, or if the antenna complexes provide too much energy in drought conditions, there may be an increased generation of reactive oxygen species, and consequently irreversible degradation of the components of the photosynthetic apparatus [21]. The resulting situation forces the plant to various processes of scattering the excess energy absorbed by chlorophyll, and one of them is increased fluorescence. The fluorimetric method allows to estimate the efficiency of the energy conversion process of PAR in the reactions of the light phase of photosynthesis [22].

There were no significant differences in the quantum photochemical reaction efficiency in PSII (Y) andETR between cultivars in control plants at PAR = 800 µmol m^−2^ s^−1^, while at PAR = 400 µmol m^−2^ s^−1^ the differences were small. However, differences were observed in plants as a result of drought stress. In the *KWS Olof* variety, the decrease in Y and ETR parameters as a result of drought was 52% and 40.9% at PAR = 800 µmol m^−2^ s^−1^ and 39% and 37.2% at PAR = 400 µmol m^−2^ s^−1^, while in the *Nokia* variety 34.6% and 37.5%, respectively, at PAR = 800 µmol m^−2^ s^−1^ and 28.6% and 25.2% at PAR = 400 µmol m^−2^ s^−1^. The *Nokia* variety was, therefore, characterized by a smaller decrease in Y and ETR, as well as higher values of both parameters in plants grown under control conditions.

In drought conditions, the *KWS Olof* variety showed a smaller decrease in CO_2_ assimilation and transpiration and higher values of these parameters at both light intensities (Table 2). Only the *KWS Olof* variety increased stress as a result of increased light intensity. It can be concluded that the *KWS Olof* variety is more resistant to drought stress because it has retained a greater ability to assimilate CO_2_ in conditions of water scarcity. This may be due to the greater ability of this variety to regulate heat dissipation (NPQ). The simultaneous reduction of Y and ETR could have been a plant defence response to drought stress. During drought stress, the stomata are closed and the efficiency of CO_2_ assimilation decreases, which reduces the consumption of ATP and NADPH and, as a consequence, slows the transport of electrons through photocircuits [23] and the Yied parameter. According to Zlatev [24], a significant decrease in the Y parameter, can be considered an indicator of the physiological regulation of electron transport by increasing the quenching process of excitation energy in PSII antennas and indicates the occurrence of photoinhibition. There was also a significant increase in non-photochemical quenching (NPQ) associated with thermal losses. In plants of the *KWS Olof* variety, NPQ increase due to drought at PAR = 800 µmol m^−2^ s^−1^ was 37%, while in the *Nokia* variety 79%. However, at PAR = 400 µmol m^−2^ s^−1^, a decrease in NPQ due to drought was noted in both varieties. Additionally, in the research of Oukarroum et al. [25], differences between spring barley cultivars in the values of chlorophyll A fluorescence parameters in response to drought stress were noted.

Chlorophyll A fluorescence parameters after dark plant adaptation (*F*_0_, *Fm* and *Fv*/*Fm*) did not differ between the tested plants. It can, therefore, be concluded that both light intensity and drought stress did not cause significant damage to Photosystem II. 

### 2.2. Antioxidant Activity

Antioxidant activity was tested by three different methods (Table 3)—DPPH, FRAP and ABTS. Results from the DPPH method in the *KWS Olof* variety ranged from 3.43 to 3.87 mg TEAC per g and from 0.95 to 2.00 mg TEAC per g in the *Nokia* variety. While the period of watering, as well as photosynthesis active radiation, had no significant influence on the antioxidant activity by this method in *KWS Olof*, in *Nokia* these factors had influence, especially under the drought condition, which caused an increase of antioxidant activity. The obtained results from the FRAP method ranged from 12.34 to 19.10 mmol TEAC per g in *KWS Olof* and from 11.71 to 18.58 mmol TEAC per g in *Nokia*. In both tested varieties drought as an abiotic stress caused an increase of activity in comparison to control samples. The same tendency was also observed in intensive irradiation, in which PAR = 800 µmol m^−2^ s^−1^ caused increasing antioxidant activity in both varieties. This increasing can be explained by antioxidant enzyme activity, such as superoxide dismutase, catalase and peroxidase, which generally increased significantly in plants under stress [26]. Results from the ABTS method in *KWS Olof* ranged from 64.43 to 66.66 mg TEAC per g and from 63.76 to 65.58 mg TEAC per g. Table 4 shows that, for the antioxidant activity tested by the ABTS method, the variety and abiotic stress, like drought and intensive irradiation, have no significant influence in comparison to DPPH and FRAP methods. Differences between the antioxidant activity was also observed in study Pérez-Labrada et al. [27] which observed antioxidant activity of tomato plants under abiotic stress (salinity). DPPH and ABTS behaved differently in the plants: antioxidant activity was decreased in those exposed to abiotic stress (−1.2%) while the activity by ABTS increased (4.7%). Ahmed et al. [28] reported the increment of antioxidant capacity (FRAP) with increasing abiotic stress (salinity) in barley. In contrast, Neffati et al. [29] found a decrement in antioxidant activity (DPPH) with increasing abiotic stress (NaCl concentrations) in coriander.

Total phenolic content (Table 4) in tested samples of variety *KWS Olof* ranged from 5.00 to 8.16 mg GAE per g and in variety *Nokia* from 5.09 to 6.38 mg GAE per g. While drought conditions caused a decrease of total phenolic content in *KWS Olof*, radiation, especially PAR 800 µmol m^−2^ s^−1^, caused an increase of these compounds in comparison to PAR = 400 µmol m^−2^ s^−1^. In *Nokia* the period of watering, as well as photosynthesis active radiation, had no the significant influence. In the study by Sarker et al. [30] abiotic stress (salinity) in *Amaranthus tricolor* leaves observed a higher amount of total phenolic content in comparison to control variant without stress. Total phenolic acid content in tested samples of *KWS Olof* ranged from 0.60 to 0.84 mg CAE per g and in *Nokia* from 0.75 to 1.65 mg CAE per g. While in *KWS Olof* significant differences were not observed, in *Nokia*, radiation PAR = 400 µmol m^−2^ s^−1^ caused an increase of total phenolic acid content in comparison to PAR = 800 µmol m^−2^ s^−1^ in control and drought cultivation conditions. From our findings it is noticeable that the biological activity of the tested samples is very strongly influenced not only by the cultivation conditions but also with the variety.

### 2.3. Antimicrobial Activity

The antimicrobial activity of green barley extract is shown in Table 5. The best antimicrobial activity of GB extracts was found against Gram-positive bacteria. The lowest antimicrobial activity of GB extract was found against Gram-negative bacteria and yeasts. The higher antimicrobial activity of GB extract was found against *B. cereus* in N/DL and against *L. monocytogenes* in N/CH and N/CL. In Boubakri et al. [31] the antimicrobial capacities of seed barley ethanolic extracts were investigated against five human pathogenic bacteria. The best antimicrobial affect was found against the growth of *S. aureus*, *M. luteus*, *E. coli*, and *S. enterica*. The strongest antimicrobial activity of barley extract was recorded against *Staphylococcus aureus* and the lowest activity was observed against *Candida albicans* [32].

### 2.4. Identification of Bioactive Compounds

**Figure 1:**
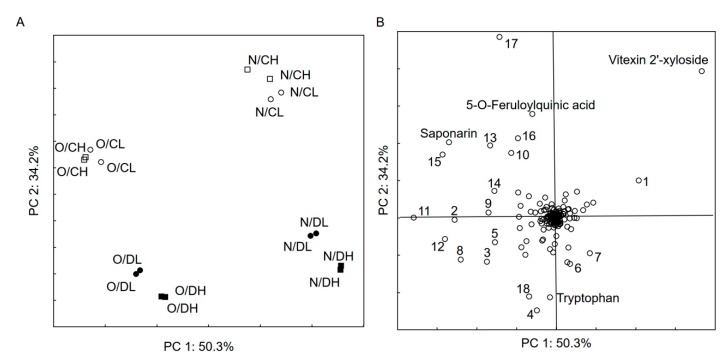
PCA, chemical profile of green barley samples determined by LC-MS. (**A**)—score plot (O—*KWS Olof*, N—*Nokia*, C—control, D—drought, L—PAR = 400, H—PAR = 800), (**B**)—loading plot (compounds—monoisotopic neutral mass_retention time: 1—522.24 Da_9.3 min, 2—372.11 Da_11.1 min, 3—400.17 Da_11.6 min, 4—304.1 Da_12 min, 5—550.3 Da_9.7 min, 6—386.12 Da_10.7 min, 7—416.17 Da_10.8 min, 8—498.23 Da_10.9 min, 9—584.23 Da_11.6 min, 10—238.08 Da_12.3 min, 11—468.22 Da_12.8 min, 12—482.24 Da_13.7 min, 13—482.24 Da_14 min, 14—524.25 Da_14.2 min, 15—568.24 Da_14.1 min, 16—524.25 Da_14.5 min, 17—568.24 Da_14.5 min, 18—414.13 Da_15.6 min).

The chemical profile of samples was compared using non-target analysis of LC-MS data collected in the negative mode. The bucket table which contains 600 compounds with intensity higher than 10^4^ cps was generated with MetaboScape 4.0. The PCA results (Figure 1) show that only a few compounds are relevant and differentiate the sample set. However, it was not possible to identify most of them using applied computational methods (CSI:FingerID and MetFrag). The score plot (Figure 1A) show clearly that chemical profile of samples not reflect to intensity of radiation, whereas factors, such as barley variety and period of watering, have an influence on the profile of synthesized compounds. Vitexin 2′-xyloside was the most abundant in *Nokia* barley, especially control samples. The non-identified compound 568.24 Da (monoisotopic neutral mass), which eluates at 14.5 min, was intensive in the control samples. The D (drought) samples were characterized by a high abundance of tryptophan and two other components (4—304.1 Da_12 min and 18—414.13 Da_15.6 min, Figure 1B).

In analysed green barleys, many compounds were identified that affect their pro-health value. The details of phenolic compounds which were identified in samples are presented in Table 6 and the results obtained are consistent with published data [33]. It has been shown that both irradiation intensity and drought stress have a significant impact on the profile of green barley polyphenolic compounds, with no simple relationship between the effect of stress factors on the content of polyphenols. Literature data indicate that the polyphenolic compounds found in barley not only have antioxidant activity but also hypolipidemic [9,34] and anti-ulcerative activity [35], and some authors also indicate anti-inflammatory or anti-cancer effects [9,36].

The phenolic profile of samples and categorical variables, such as period of watering, intensity of radiation and barley variety, were included during k-means clustering with the V-fold cross-validation method. This statistical procedure was performed to show which cultivation factors have an influence on phenolic components. The results are presented in Table 7 and two clusters were generated. It was noticed that only the period of watering had a discriminative power (*p* < 0.05), while other cultivation variables were not relevant. The most discriminative phenolic compounds (the highest F value) were sinapic acid, ferulic acid and sinapoyl-beta-glucose. However, saponarin, vicetin-2 and vitexin 2′-xyloside dominated in samples, but they are not statistically different in clusters (*p* > 0.05).

## 3. Materials and Methods

### 3.1. Materials

Barley grains were purchased from KWS Lochow Polska (Prusy, Poland). Acetic acid, 2,2-difenyl-1-picrylhydrazyl (DPPH), 6-hydroxy-2,5,7,8-tetramethylchroman-2-carboxylic acid (Trolox), Folin–Ciocalteu reagent, quercetin, caffeic acid, apigenin, chlorogenic acid, vanillic acid, *p*-coumaric acid, ferulic acid, sinapic acid, quercetin, kaempferol, 2,2′-azino-bis(3-ethylbenzothiazoline-6-sulfonic acid) diammonium salt (ABTS) and 2,4,6-tris(2-pyridyl)-s-triazine (TPTZ) were purchased from Sigma-Aldrich (Saint-Louis, MO, USA). Ethanol, sodium carbonate, sallic acid, aluminium chloride, potassium acetate, hydrochloric acid, Arnova reagent and sodium hydroxide were purchased from Reachem (Bratislava, Slovakia. Potassium persulphate and ferric chloride were purchased from Avantor Performance Materials Poland (Gliwice, Poland). Dimethylsulfoxid (DMSO) was purchased from Penta (Praha, Czech Republic). Mueller–Hinton agar and sabouraud dextrose agar were purchased from Biolife (Milano, Italy). Ampicillin and gentamicin were purchased from Oxoid (Basingstoke, UK). Acetonitrile was purchased from Merck KGaA (Darmstadt, Germany). All other chemicals were purchased from Sigma-Aldrich, in their best available purity grade.

### 3.2. Plant Material and Growing Conditions

Two varieties of spring barley (*Hordeum vulgare* L.), *KWS Olof* and *Nokia*, were used in this study. Plants were grown for 18 days in potted cultures in a climate chamber (65%–75% relative humidity, 26 °C, photoperiod—16:8 h (day:night) using Fito Panels 150 (Biogenet, Józefów, Poland). The barley was sown in 2.5 dm^3^ pots filled with the same amount of soil at pH 5.5. Thirty barley seeds of equal size were sown in each pot. Soil moisture was maintained at a constant level of 12% and regularly watered (100 mL H_2_O/vase every 48 h). Two abiotic stress factors were used in the experiment: drought and intensive irradiation. Drought was imposed after nine days of cultivation by stopping the water supply. The control plants were irrigated regularly. Moreover, two levels of photosynthesis active radiation (PAR) were used throughout the growing period: 400 µmol m^−2^ s^−1^ and 800 µmol m^−2^ s^−1^ for control and drought as well. Descriptions of the analysed crop variants and the abbreviations used are presented in Table 8. After seven days of stress the physiological state of plants was determined in both control and stressed plants. Plant material for antioxidant activity estimation was collected after 18 days. Plants were frozen at −85 °C and freeze-dried. The freeze-drying process was performed using an Alpha 2–4 LD plus lyophilizer (Martin Christ, Osterode am Harz, Germany). The process was initiated with a freezing stage at −35 °C for 20 h, followed by a main drying step on a shelf in 5 °C for 12 h and then final drying up to 20 °C for 2 h. Green barley (GB) was ground, packed in polypropylene pouches and stored in a cold store.

### 3.3. Evaluation of the Physiological State of Plants

#### 3.3.1. Plant Photosynthesis

Rates of CO_2_ exchange in the leaf chamber to the photosynthesis rate (A) of single leaves were measured on the first young fully mature healthy leaf using a portable photosynthesis system (LCpro-SD, ADC BioScientific Ltd., Hoddesdon, UK) with a narrow leaf chamber (area: 5.8 cm^2^). The CO_2_ concentration (Reference CO_2_) in the leaf chamber was respectively kept at 360 vpm and the leaf chamber temperature (Tch) maintained at 26 ± 1 °C. The flow rate of air (u) was 200 µmol s^−1^. The H_2_O concentration (Reference H_2_O) and flow (u) were maintained at ambient values. Plant photosynthesis, for each combination of GB, was measured after 6-h dark adaptation of plants at 400 µmol m^−2^ s^−1^, adjusted automatically by a red–blue light-emitting diode (LED) light source (LCP Narrow Lamp, ADC BioScientific Ltd., Hoddesdon, UK). Settings protocols and methods of measurement were selected in accordance with the manufacturer’s instructions. The following parameters were measured: Photosynthesis rate—A (µmol m^−2^ s^−1^), transpiration rate—E (mmol m^−2^ s^−1^), sub-stomatal CO_2_—Ci (µmol mol^−1^) and stomatal conductance—Gs (mol m^−2^ s^−1^).

#### 3.3.2. Plant Chlorophyll Fluorescence

Chlorophyll fluorescence was measured at the same leaf as photosynthesis with a multi-mode chlorophyll fluorometer (OS5p, Opti-Sciences, Inc., Hudson, NY, USA) with a PAR clip (which allows for the measurement of PAR or PPFD and leaf temperature along with a yield test). The kinetic protocol was selected. The fluorescence kinetics test combines measurements under light conditions and measurements after dark adaptation. Fluorescence measurements were done in triplicate for each combination, 6 h after the leaves were dark-adapted. Settings for the fluorometer protocols were selected according to Sulewska et al. [37] as follows: Modulation source: red; modulation intensity: 25; detector gain: 08; saturation flash intensity: 30; flash count: 001; flash rate: 255 (s).

The following parameters were measured: *F*_0_—minimum fluorescence, *Fm*—maximum fluorescence, *Fv*—variable fluorescence and *Y*—quantum yield of photosynthetic energy.

The maximum photochemical efficiency of photosystem II (*Fv*/*Fm*) and electron transport rate (ETR) were calculated according to formulas:

$$\frac{Fv}{Fm}=\frac{Fm-F_{0}}{Fm}$$

ETR=Y·0.84·0.5·PAR

### 3.4. Ethanolic Extraction

For ethanolic extract the freeze-dried GB samples were used. A sample of 0.5 g was extracted with 40 mL of 80% ethanol for 2 h. After centrifugation at 4000× *g* (Rotofix 32 A, Hettich, Germany) for 10 min supernatants were decanted, filtered (0.22 μm) and stored in a freezer at −20 °C in a dark, glass flask. Extraction was carried out in triplicate.

### 3.5. Determination of Total Phenolic Content

The total phenolic compounds (TPC) content was determined by the Folin–Ciocalteu colorimetric method [38]. A 0.1 mL sample was mixed with 0.1 mL of the Folin–Ciocalteu reagent, 1 mL of 20% (*w/v*) sodium carbonate, and 8.8 mL of distilled water and left in in darkness for 30 min. The absorbance at 700 nm was measured using the spectrophotometer Jenway (6405 UV/VIS, Stone, UK). Gallic acid (25–300 mg/L; *R*^2^ = 0.998) was used as a standard and the results were expressed in mg/g of gallic acid equivalent (GAE).

### 3.6. Determination of Total Phenolic Acid Content

Total phenolic acids (TPA) content was determined using the method of Polish Farmakopea [39]. A 0.5 mL sample extract was mixed with 0.5 mL of 0.5 M hydrochloric acid, 0.5 mL of Arnova reagent (10% NaNO_2_ + 10% Na_2_MoO_4_), 0.5 mL of 1 M sodium hydroxide (*w/v*) and 0.5 mL of water. Absorbance at 490 nm was measured using a Jenway spectrophotometer (6405 UV/VIS, Stone, UK). Caffeic acid (1–200 mg/L, *R*^2^ = 0.999) was used as a standard and the results were expressed in mg/g of caffeic acid equivalent.

### 3.7. Determination of Antioxidant Activity

#### 3.7.1. Free Radical Scavenging Activity Using ABTS

ABTS radical cation decolourization assay was determined by the method of Re et al. [40] with slight modifications. ABTS (2,2′-azinobis [3ethylbenzthiazoline]-6-sulfonic acid) was dissolved in distilled water to 7 mM concentration, and potassium persulphate was added to achieve a concentration of 2.45 mM. The reaction mixture was left at room temperature overnight (12–16 h) in the dark before use. The resultant intensely-coloured ABTS^•+^ radical cation was diluted with 0.01 M PBS (phosphate buffered saline), pH 7.00, to give an absorbance value of ~0.70 at 734 nm. Two millilitres of ABTS solution was mixed with 0.98 mL of PBS and 0.02 mL of sample. Absorbance was measured spectrophotometrically (Jenway 6405 UV/VIS, Stone, UK) 6 min. after the addition of the sample. Trolox (100–1000 mg/L; *R*^2^ = 0.9991) was used as a standard, and the results were expressed in mg/g of Trolox equivalents.

#### 3.7.2. Ferric Reducing Antioxidant Power

Ferric reducing antioxidant power (FRAP) was analysed according to the procedure method described by Benzie and Strain [41]. Nine microlitres of PJPC extract was added to 270 µL of TPTZ solution (10 mM TPTZ, 20 mM ferric chloride and 300 mM sodium acetate buffer (pH 3.6) at a ratio of 1:1:10 (*v/v/v*)) in 96-well plates. After 6 min of incubation at 37 °C, absorption was measured at 595 nm in a UV–VIS spectrophotometer (Multiskan GO, Thermo Fisher Scientific, Vantaa, Finland). The results of antioxidant activities were presented as Trolox equivalent antioxidant capacity (TEAC) per 1 g of dry matter of the examined material. 

#### 3.7.3. Free Radical Scavenging Activity Using DPPH

Radical scavenging activity of extracts was measured using 2,2-diphenyl-1-picrylhydrazyl (DPPH) [42]. The sample (0.4 mL) was mixed with 3.6 mL of DPPH solution (0.025 g DPPH in 100 mL methanol). Absorbance of the reaction mixture was determined using the Jenway spectrophotometer (6405 UV/VIS, Stone, UK) at 515 nm. Trolox (6-hydroxy-2,5,7,8-tetramethylchroman-2-carboxylic acid) (10–100 mg/L; *R*^2^ = 0.989) was used as the standard and the results were expressed in mg/g Trolox equivalents.

### 3.8. Antimicrobial Activity

For the antimicrobial assays, the plant extracts were dissolved in 1% dimethylsulfoxid (DMSO). Stock solutions of plant extracts were stored at −16 °C in a refrigerator until the experiments were initiated. For the experiment, blank paper discs (Oxoid, UK) were impregnated with plant extract.

#### 3.8.1. Microorganisms

Nine strains of microorganisms were tested, including three Gram-negative bacteria (*Escherichia coli* CCM 3988, *Salmonella enterica* subsp. *enterica* CCM 3807, *Yersinia enterocolitica* CCM 5671), three Gram-positive bacteria (*Bacillus cereus* CCM 2010, *Listeria monocytogenes* CCM 4699, *Staphylococcus aureus* subsp. *aureus* CCM 4223) and three yeasts (*Candida albicans* CCM 8186, *Candida glabrata* CCM 8270, *Candida tropicalis* CCM 8223). Tested bacteria and yeast strains were collected from the Czech Collection of Microorganisms (Brno, Czech Republic). The bacterial strains were grown on Mueller–Hinton agar plates at 37 °C, whereas the yeasts were grown in Sabouraud dextrose agar at a temperature of 28 °C. The stock cultures were maintained at a temperature of 4 °C.

#### 3.8.2. Detection of Antimicrobial Activity

Antimicrobial activity of each plant extract was determined by a disc diffusion method. Briefly, a quantity of 100 μL of suspension of the test bacteria were grown in 10 mL of fresh media until the concentration of 10^5^ CFU/mL was reached. Then, 100 μL of the microbial suspension was spread onto Mueller–Hinton agar and impregnated discs were placed onto the surface of the inoculated agar. After incubation at 37 °C and 28 °C for 24 h, the diameters of the inhibition zones were measured. All measurements were to the closest whole millimetre. Each antimicrobial assay was performed in, at least, triplicate. Filter discs impregnated with a 10 μL of distilled water were used as a negative control. As positive control blank discs were impregnated with ampicillin (10 µg/disc) for Gram-positive bacteria and yeasts and gentamicin (10 µg/disc) for Gram-negative bacteria. Experiments were run in triplicate.

### 3.9. LC-MS Analysis

Reversed-phase (C18 column) ultra-high-performance liquid chromatography electrospray ionization mass spectrometry (RP-UHPLC-ESI-MS) analysis was performed using a Dionex UltiMate 3000 UHPLC (Thermo Fisher Scientific, Sunnyvale, CA, USA) coupled to a Bruker maXis impact ultrahigh resolution orthogonal quadrupole-time-of-flight accelerator (qTOF) equipped with an ESI source and operated in the negative-ion mode (Bruker Daltonik, Bremen, Germany). The RP chromatographic separation was achieved with a Synergi 4 µm Fusion-RP 80 Å, LC column 150 × 3.0 mm (Phenomenex, Torrance, CA, USA). The mobile phase was composed of water containing 0.1% acetic acid (A) and solution of 0.1% acetic acid in acetonitrile/water (95/5 *v/v*) (B). The flow rate was 0.3 mL/min with a gradient elution of 1–100% B over 20 min. The column temperature was set to 40 °C. The syringe and needle were washed before and after injection of each samples (water:methanol, 1:1 (*v/v*)). The carryover between samples was not observed. The ESI-MS settings were previously described by Mildner-Szkudlarz et al. [43]. Molecular ions: [M − H]^−^ for phenolic compounds were extracted from full scan chromatograms (±0.005 *m/z*) and peak areas were integrated with QuantAnalysis 2.1 (Bruker Daltonik, Bremen, Germany). The compounds present in each sample were identified based on the retention time of the standard and/or the molecular mass and structural information from the MS detector during the MS/MS experiments. The limit of quantification (LOQ where S/N >15) was determined for caffeic acid, apigenin, chlorogenic acid, vanillic acid, *p*-coumaric acid, ferulic acid, sinapic acid, quercetin and kaempferol and it was not lower than 0.01 µg/mL.

The tandem mass spectrometric data were used for searching the molecular structure using two computational methods. We used CSI:FingerID, which combines fragmentation tree computation and machine learning [44,45] and the in silico fragmenter MetFrag [46]. The possible molecular formulae for a MS precursor ion of a not-identified (NI) compound was calculated with the SmartFormula3D (Bruker, Bremen, Germany) command by combining two spectra, MS and MS/MS.

### 3.10. Statistical Analysis

All measurements were repeated in triplicate, unless stated otherwise. One-way analysis of variance (ANOVA) was carried out independently for each dependent variable. The post-hoc Tukey HSD multiple comparison test was used to identify statistically homogeneous subsets at α = 0.05. The principal component analysis (PCA) was performed to compare the chemical profile of samples. The k-means algorithm with the V-fold cross-validation method was used to discriminate samples according to the phenolic profile of samples and planting strategy. Statistical analysis was performed with Statistica 13 software (Dell Software Inc., Round Rock, TX, USA). The LC-MS data were imported and processed and a bucket table (the collected compounds’ signals of samples as a matrix) was generated from the mass calibrated and retention time aligned data using MetaboScape 4.0 (Bruker, Bremen, Germany).

## 4. Conclusions

Abiotic stress in the form of water deficiency in both varieties caused a significant decrease in CO_2_ assimilation, however, at a different level. *KWS Olof* was characterized by lower CO_2_ assimilation under control conditions, but retained higher levels under drought stress. Smaller, but significant, differences between varieties were noted in the transpiration level. It can be concluded that the *KWS Olof* variety is more resistant to drought stress because it retained a greater ability to assimilate CO_2_ in conditions of water scarcity. This may be due to the greater ability of this variety to regulate heat dissipation (NPQ). Chlorophyll A fluorescence parameters after dark plant adaptation (*F*_0_, *Fm* and *Fv*/*Fm*) did not differ between the tested plants. It can, therefore, be concluded that both light intensity and drought stress did not cause significant damage to Photosystem II. In both tested varieties drought as an abiotic stress caused an increase of antioxidant activity in comparison to control samples. The same tendency was also observed in intensive irradiation, in which PAR = 800 µmol m^−2^ s^−1^ caused increasing antioxidant activity in both varieties. From our findings it is visible that the biological activity of the tested samples is very strongly influenced not only by the cultivation condition but also with variety. The results clearly show that the chemical profile of the samples does not reflect the intensity of radiation, whereas factors, such as barley variety and period of watering, have an influence on the profile of synthesized compounds.

## Figures and Tables

**Table 1:** The results of the analysis of plant photosynthesis (PAR = 400 µmol m^−2^ s^−1^).

Sample	Ci (µmol mol^−1^)	E (mmol m^−2^ s^−1^)	Gs (mol m^−2^ s^−1^)	A (µmol m^−2^ s^−1^)
O/CH	280.7 ± 2.5 ^a^	3.783 ± 0.399 ^ab^	0.263 ± 0.049 ^ab^	10.910 ± 1.495 ^b^
O/CL	275.7 ± 3.5 ^a^	3.093 ± 0.467 ^ab^	0.197 ± 0.047 ^b^	8.693 ± 1.522b ^c^
O/DH	183.3 ± 18.2 ^c^	0.817 ± 0.407 ^d^	0.030 ± 0.020 ^cd^	3.533 ± 1.780 ^de^
O/DL	232.0 ± 23.9 ^abc^	1.983 ± 0.556 ^c^	0.103 ± 0.041 ^c^	7.090 ± 1.453 ^cd^
N/CH	257.7 ± 3.0 ^ab^	3.947 ± 0.110 ^a^	0.293 ± 0.015 ^a^	15.537 ± 1.058 ^a^
N/CL	255.0 ± 6.1 ^ab^	2.920 ± 0.056 ^bc^	0.220 ± 0.036 ^ab^	11.787 ± 1.170 ^b^
N/DH	212.0 ± 37.0 ^bc^	0.370 ± 0.135 ^d^	0.010 ± 0.010 ^d^	1.303 ± 0.664 ^e^
N/DL	233.0 ± 26.2 ^abc^	0.463 ± 0.112 ^d^	0.017 ± 0.005 ^cd^	1.443 ± 0.601 ^e^

^a–e^ different letters in rows indicate statistically different mean values (*p* < 0.05). Ci—sub-stomatal CO_2_; E—transpiration rate; Gs—stomatal conductance; A—photosynthesis rate.

**Table 2:** Chlorophyll fluorescence results.

Sample	Y	NPQ	ETR	F_0_	Fm	Fv/Fm
O/CH	0.252 ± 0.026 ^a^	1.530 ± 0.130 ^abc^	35.17 ± 3.87 ^a^	255.0 ± 3.6 ^a^	1117 ± 25 ^ab^	0.771 ± 0.015 ^ab^
O/CL	0.181 ± 0.004 ^bc^	1.967 ± 0.015 ^ab^	25.03 ± 0.56 ^bc^	261.0 ± 8.6 ^a^	1174 ± 55 ^a^	0.773 ± 0.009 ^ab^
O/DH	0.120 ± 0.021 ^d^	2.100 ± 0.409 ^a^	20.80 ± 2.13 ^cd^	253.0 ± 35.0 ^a^	1047 ± 107 ^ab^	0.756 ± 0.024 ^b^
O/DL	0.113 ± 0.003 ^d^	1.507 ± 0.289 ^abc^	15.73 ± 0.50 ^d^	264.7 ± 20.0 ^a^	1076 ± 16 ^ab^	0.766 ± 0.015 ^ab^
N/CH	0.264 ± 0.007 ^a^	0.920 ± 0.242 ^c^	36.83 ± 1.07 ^a^	236.3 ± 8.3 ^a^	1111 ± 14 ^ab^	0.787 ± 0.007 ^a^
N/CL	0.206 ± 0.022 ^b^	1.593 ± 0.320 ^abc^	28.23 ± 2.77 ^b^	246.3 ± 12.2 ^a^	1128 ± 52 ^ab^	0.781 ± 0.002 ^ab^
N/DH	0.165 ± 0.015 ^bc^	1.950 ± 0.134 ^ab^	23.00 ± 2.17 ^bc^	223.3 ± 3.2 ^a^	1095 ± 29 ^ab^	0.776 ± 0.040 ^a^
N/DL	0.152 ± 0.006 ^cd^	1.280 ± 0.204 ^bc^	21.13 ± 0.68 ^cd^	228.7 ± 4.0 ^a^	999 ± 41 ^b^	0.782 ± 0.011 ^ab^

^a–d^ different letters in columns indicate statistically different mean values (*p* < 0.05). Y—quantum yield of photosynthetic energy; NPQ—non-photochemical quenching; ETR—electron transport rate; F_0_—minimum fluorescence; Fm—maximum fluorescence; Fv/Fm—maximum photochemical efficiency of photosystem II.

**Table 3:** Antioxidant activity of GB.

Parameter	DPPH [mg g^−1^]	FRAP [mmol g^−1^]	ABTS [mg g^−1^]
O/CH	3.87 ± 0.37 ^a^	18.67 ± 0.64 ^a^	64.43 ± 1.17 ^bc^
O/CL	3.54 ± 0.21 ^a^	12.34 ± 0.45 ^d^	65.92 ± 0.96 ^ab^
O/DH	3.71 ± 0.20 ^a^	19.10 ± 1.47 ^a^	66.46 ± 1.15 ^a^
O/DL	3.43 ± 0.52 ^a^	14.50 ± 0.42 ^bc^	66.66 ± 0.29 ^a^
N/CH	0.95 ± 0.33 ^d^	16.50 ± 2.62 ^b^	65.58 ± 0.43 ^b^
N/CL	1.20 ± 0.45 ^c^	11.71 ± 1.09 ^d^	65.38 ± 0.96 ^b^
N/DH	2.00 ± 0.64 ^b^	18.58 ± 1.75 ^a^	65.38 ± 1.68 ^ab^
N/DL	1.34 ± 0.25 ^bc^	13.27 ± 1.27 ^c^	63.76 ± 1.10 ^c^

^a–d^ different letters in columns indicate statistically different mean values (*p* < 0.05).

**Table 4:** Total phenolic acids and total phenolic compounds in GB.

Parameter	TPA [mg g^−1^]	TPC [mg g^−1^]
O/CH	0.84 ± 0.07 ^b^	8.16 ± 0.50 ^a^
O/CL	0.60 ± 0.03 ^d^	6.87 ± 1.07 ^b^
O/DH	0.78 ± 0.07 ^c^	6.60 ± 1.57 ^b^
O/DL	0.78 ± 0.01 ^c^	5.00 ± 0.94 ^c^
N/CH	0.75 ± 0.03 ^c^	5.09 ± 1.07 ^c^
N/CL	1.27 ± 0.28 ^a^	6.38 ± 0.63 ^b^
N/DH	0.81 ± 0.04 ^b^	6.16 ± 0.69 ^b^
N/DL	1.65 ± 1.23 ^a^	5.67 ± 0.38 ^c^

^a–d^ different letters in columns indicate statistically different mean values (*p* < 0.05). TPA—total phenolic acids; TPC—total phenolic compounds.

**Table 5:** Antimicrobial activity of GB.

Microorganisms	O/CH	O/CL	O/DH	O/DL	N/CH	N/CL	N/DH	N/DL
*E. coli*	7.67 ± 0.58	7.67 ± 0.58	8.33 ± 0.58	7.33 ± 0.58	8.33 ± 0.15	7.33 ± 0.58	7.33 ± 0.58	7.67 ± 0.58
*S. enetrica* subsp. *enterica*	7.67 ± 0.58	7.33 ± 0.58	6.33 ± 0.58	6.67 ± 1.53	8.67 ± 0.58	7.33 ± 1.53	6.67 ± 0.58	7.67 ± 0.58
*Y. enterocolitica*	7.67 ± 0.58	7.67 ± 0.58	7.33 ± 0.58	6.67 ± 0.58	7.67 ± 0.58	6.33 ± 0.58	6.67 ± 0.58	7.67 ± 0.58
*B. cereus*	10.33 ± 0.58	8.67 ± 0.58	9.67 ± 1.15	10.67 ± 1.53	9.33 ± 0.58	10.33 ± 0.58	10.67 ± 0.58	11.33 ± 0.58
*L. monocytogenes*	10.33 ± 0.58	10.33 ± 0.58	10.67 ± 1.15	8.33 ± 1.15	11.33 ± 0.58	11.33 ± 1.15	10.67 ± 0.58	10.67 ± 1.15
*S. aureus*	8.67 ± 0.58	9.33 ± 0.58	10.33 ± 0.58	10.67 ± 0.58	8.67 ± 0.58	9.67 ± 0.58	8.67 ± 0.58	9.33 ± 0.58
*C. albicans*	6.33 ± 0.58	7.67 ± 0.58	7.33 ± 0.58	7.33 ± 1.15	7.67 ± 1.15	6.67 ± 0.58	5.67 ± 0.58	7.67 ± 0.58
*C. glabrata*	7.33 ± 0.58	7.33 ± 0.58	5.67 ± 0.58	6.67 ± 0.58	6.33 ± 0.58	6.33 ± 0.58	7.67 ± 0.58	6.67 ± 0.58
*C. tropicalis*	6.33 ± 0.58	6.67 ± 0.58	6.33 ± 0.58	7.33 ± 0.58	6.67 ± 0.58	5.67 ± 0.58	6.67 ± 1.15	5.67 ± 1.15

**Table 6:** Total phenolic content (GEA, mg/g DM) and phenolic compounds determined by LC-MS (μg/g DM).

Variable/Sample *	O/CH	O/CL	O/DH	O/DL	N/CH	N/CL	N/DH	N/DL
1-O-Sinapoyl-beta-d-glucose **	12.14 ^b^	4.52 ^a^	41.45 ^d^	46.83 ^e^	12.93 ^b^	11.60 ^b^	34.57 ^c^	41.19 ^d^
4-Hydroxybenzoic acid	0.59 ^cd^	0.49 ^b^	0.51 ^bc^	0.44 ^b^	0.58 ^cd^	0.62 ^d^	0.34 ^a^	0.46 ^b^
Tricin **	4.94 ^f^	1.62 ^b^	5.28 ^f^	3.50 ^e^	2.50 ^d^	0.88 ^a^	3.22 ^e^	2.05 ^c^
Apigenin	0.12 ^d^	<LOD	0.09 ^c^	0.09 ^c^	0.12 ^d^	0.06 ^b^	0.13 ^d^	0.05 ^b^
Apigenin 7-alpha-l-arabinopyranosyl-(1->6)-glucoside **	6.66 ^d^	5.59 ^bc^	5.75 ^c^	5.49 ^b^	6.56 ^d^	5.58 ^bc^	5.43 ^b^	4.57 ^a^
Ferulic acid	0.94 ^c^	0.45 ^ab^	3.62 ^e^	0.99 ^c^	0.66 ^b^	0.24 ^a^	2.50 ^d^	0.35 ^a^
Vitexin 2′-xyloside **	1.38 ^a^	0.56 ^a^	0.68 ^a^	0.53 ^a^	109.78 ^e^	80.12 ^d^	45.19 ^c^	27.72 ^b^
Vicetin-2 **	75.83 ^a^	77.26 ^a^	78.76 ^ab^	76.23 ^a^	84.92 ^b^	101.60 ^c^	94.14 ^bc^	87.52 ^abc^
Lutonarin **	24.30 ^h^	13.79 ^g^	8.82 ^c^	9.51 ^d^	10.94 ^e^	11.82 ^f^	6.34 ^b^	5.26 ^a^
Rutin	<LOD	<LOD	<LOD	<LOD	<LOD	<LOD	3.88	<LOD
Saponarin **	179.80 ^d^	157.39 ^c^	145.51 ^c^	112.78 ^b^	149.35 ^c^	125.92 ^b^	62.50 ^a^	49.72 ^a^
Sinapic acid	3.37 ^b^	2.10 ^a^	40.65 ^f^	23.67 ^e^	5.15 ^c^	2.02 ^a^	41.64 ^f^	17.12 ^d^
Vanillic acid	1.47 ^a^	2.04 ^d^	1.97^bcd^	1.90 ^bcd^	1.67 ^ab^	2.06 ^d^	1.77 ^bc^	1.91 ^bcd^
*p*-coumaric acid	0.19 ^a^	0.15 ^a^	0.62 ^b^	0.19 ^a^	<LOD	<LOD	0.15 ^a^	<LOD

* sinapoyl-beta-d-glucose calculated as sinapic acid, tricin as luteolin, apigenin 7-alpha-l-arabinopyranosyl-(1->6)-glucoside, vitexin 2′-xyloside, vicetin-2, lutonarin, saponarin were calculated as apigenin. ** compound identified using tandem mass spectrometric data and CSI:FingerID and MetFrag computational methods. ^a–h^ different letters in rows indicate statistically different mean values (*p* < 0.05).

**Table 7:** Results of k-means clustering algorithm, the number of clusters was determined automatically from the data by using the V-fold cross-validation method.

**ANOVA for Continuous Variables**				
**Compound**	**Means ± SD (µg/g)**		**F**	***p* Value**
	**1st Cluster**	**2nd Cluster**		
Sinapic acid *	5.95 ± 6.01	35.32 ± 9.05	61.71	0.0000
Ferulic acid *	0.53 ± 0.26	2.37 ± 1.19	23.24	0.0003
Sinapoyl-beta-glucose *	16.48 ± 13.41	40.95 ± 5.59	17.72	0.0009
*p*-coumaric acid *	0.07 ± 0.09	0.32 ± 0.23	9.51	0.0081
Tricin *	2.40 ± 1.45	4.00 ± 1.00	5.57	0.0333
Rutin	0.00 ± 0.00	1.29 ± 2.00	4.37	0.0552
Apigenin	0.07 ± 0.05	0.11 ± 0.02	3.38	0.0874
4-Hydroxybenzoic acid	0.55 ± 0.07	0.43 ± 0.08	9.12	0.0092
Lutonarin	13.22 ± 6.56	8.22 ± 1.49	3.29	0.0912
Vitexin 2′-xyloside	43.91 ± 46.24	15.46 ± 23.04	1.94	0.1853
Saponarin	132.44 ± 47.39	106.93 ± 37.44	1.25	0.2815
Apigenin 7-alpha-l-arabinopyranosyl-(1->6)-glucoside	5.79 ± 0.81	5.56 ± 0.15	0.48	0.4981
Vicetin-2	85.43 ± 10.00	83.04 ± 9.50	0.22	0.6459
Vanillic acid	1.83 ± 0.24	1.88 ± 0.11	0.19	0.6721
**Independence Test for Categorical Variables**				
**Variable**	Frequency **			
	**1st Cluster (Samples/Total)**	**2nd Cluster (Samples/Total)**	**Chi-Square**	***p* Value**
Watering *	C(4/8), D(0/8)	C(0/8), D(4/8)	9.60	0.0019
PAR	L(3/8), H(2/8)	L(1/8), H(2/8)	1.07	0.3017
Variety	O(2/8), N(3/8)	O(2/8), N(1/8)	1.07	0.3017

* the significant variables (*p* < 0.05) which discriminate samples into two groups. ** total number of planting variant is 8 according to Table 1, for example, the notation C(4/8) means that 50% control samples were collected in 1st cluster (O—*KWS Olof*, N—*Nokia*, C—control, D—drought, L—PAR = 400, H—PAR = 800).

**Table 8:** Description of sample ID and cultivation conditions.

Sample ID	Variety	Control/Drought	Photosynthesis Active Radiation (µmol m^−2^ s^−1^)
O/CH	*KWS Olof*	Control	800
O/CL	*KWS Olof*	Control	400
O/DH	*KWS Olof*	Drought	800
O/DL	*KWS Olof*	Drought	400
N/CH	*Nokia*	Control	800
N/CL	*Nokia*	Control	400
N/DH	*Nokia*	Drought	800
N/DL	*Nokia*	Drought	400
